# Sex-Specific Epigenetic Patterns in Endocannabinoid System Genes Following High-Altitude Exposure: An Exploratory Study

**DOI:** 10.3390/brainsci16050500

**Published:** 2026-05-02

**Authors:** Carlotta Marrangone, Alessio Mosca, Manuel Marzola, Francesca Martella, Martina Di Bartolomeo, Vittore Verratti, Giovanni Martinotti, Claudio D’Addario

**Affiliations:** 1 Department of Neuroscience, Imaging and Clinical Sciences, “G. d’Annunzio” University, 66100 Chieti, Italy; carlotta.marrangone@gmail.com (C.M.); alessio.mosca909@gmail.com (A.M.); 2 Department of Medicine and Aging Sciences, “G. d’Annunzio” University, 66100 Chieti, Italy; manuel.marzola@phd.unich.it; 3 Department of Bioscience and Technology for Food, Agriculture and Environment, University of Teramo, 64100 Teramo, Italy; fmartella@unite.it (F.M.); mdibartolomeo@unite.it (M.D.B.); cdaddario@unite.it (C.D.); 4 Department of Science, “G. d’Annunzio” University, Italian Society of Mountain Medicine, 66100 Chieti, Italy; vittore.verratti@unich.it

**Keywords:** altitude, endocannabinoid system, epigenetic, sex differences, psychological distress

## Abstract

**Highlights:**

**What are the main findings?**

- High-altitude related stress may be associated with sex-specific epigenetic differences in the endocannabinoid system, with male-specific increases in *CNR1* promoter methylation.
- Changes in *CNR1* methylation and stress-related miRNA expression suggest a modulation of CB1 signaling during extreme environmental exposure.

**What are the implications of the main findings?**

- *CNR1* CpG4 methylation represents an exploratory candidate for future biomarker investigation, pending validation.
- The observed sex-dependent associations with depressive symptoms underscore the need for sex-informed models of resilience and vulnerability to environmental stress.

**Abstract:**

**Background/Objectives**: High-altitude exposure represents a complex psychophysiological stressor involving hypoxia, physical effort, sleep disruption and psychological strain. The endocannabinoid system (ECS) plays a key role in stress regulation, yet its epigenetic modulation under extreme environmental conditions remains poorly characterized. This pilot and exploratory study investigated DNA methylation and descriptive microRNA (miRNA) expression patterns of *CNR1* and *FAAH* genes, and their associations with mood and anxiety outcomes, in trekkers exposed to Himalayan high altitude. **Methods**: Twenty-one healthy lowlanders completed a longitudinal expedition from 2860 m to 5050 m. Psychometric measures (SVARAD, BDI, SAS, SHAPS) and saliva samples were collected at baseline (T0) and at high altitude (T1). DNA methylation of *CNR1* and *FAAH* regulatory regions was quantified by pyrosequencing. Exosomal miRNAs targeting these genes were profiled using qRT-PCR, on pooled samples; results are presented descriptively. **Results**: DNA methylation analysis revealed heterogeneous, sex-specific epigenetic patterns following high-altitude exposure. A significant increase in *CNR1* promoter methylation at CpG4 was observed in males at T1, whereas methylation remained largely stable in females. Descriptive miRNA expression data showed bidirectional differences between groups, consistent with context-dependent stress regulation. Convergent directional patterns between miR-23b-3p expression and *CNR1* methylation in males were observed. However, given the descriptive nature of the miRNA data, this observation is purely exploratory and requires replication before any mechanistic conclusions can be drawn. Psychometrically, participants showed a mild mood decline without overt clinical symptoms. Sex-specific differences in the relationship between *CNR1* methylation and psychometric outcomes were observed and warrant further investigation in adequately powered cohorts. **Conclusions**: These preliminary findings suggest that *CNR1* epigenetic regulation warrants further investigation as a potential indicator of stress adaptation and psychological responses and underscore the need to consider sex differences when evaluating resilience and vulnerability to extreme environments.

## 1. Introduction

High-altitude mountaineering represents an extreme physical and psychological challenge that combines multiple stressors, including hypoxia, cold exposure, physical exhaustion, sleep fragmentation and psychological pressure [1,2,3]. While many individuals successfully complete such expeditions, others experience significant psychological distress, raising questions about individual susceptibility and the biological mechanisms underlying stress responses in extreme environments. Understanding these individual differences is critical not only for optimizing climber safety and performance but also for identifying those who may require additional support or modified protocols. Several recent overviews summarize molecular, neurophysiological and clinical evidence suggesting that acute and chronic altitude exposure can alter neurotransmitter systems, cerebral perfusion and neural network function, with downstream effects on cognition and affect [4]. Recent evidence underscores the psychological burden associated with high-altitude exposure. Large-scale prospective data from the Intern Health Study demonstrated that individuals living at higher elevations reported a significantly increased risk of depression and anxiety compared to those at sea level, suggesting a dose–response relationship between altitude and mood disturbance [5]. In line with these findings, a recent systematic review and meta-analysis reported that the prevalence of depressive symptoms is substantially higher in high-altitude populations, with heterogeneity across studies but a consistent overall signal of increased vulnerability [6]. The role of acute mountain sickness (AMS) in mediating these affective outcomes has also been highlighted. A study identified anxiety, irritability and depression as both predictors and correlates of AMS severity, suggesting a bidirectional relationship between somatic symptoms of altitude illness and psychological distress [7]. Nevertheless, despite accumulating epidemiological and clinical evidence, targeted field studies using standardized psychometric tools remain relatively scarce, and the neurobiological mechanisms underlying these changes are only beginning to be explored [7].

The endocannabinoid system (ECS) plays a crucial role in stress regulation, mood modulation, and adaptation to environmental challenges, such as hypoxia and high-altitude exposure [8,9]. Two key components of this system are the cannabinoid receptor type 1 (*CNR1*/CB1) and fatty acid amide hydrolase (FAAH), the primary enzyme responsible for degrading anandamide, the brain’s endogenous cannabinoid. Variations in the activity of these genes have been associated with individual differences in stress resilience, anxiety responses, and psychological well-being [10,11,12]. Importantly, the ECS exhibits marked sexual dimorphism in both baseline function [13] and stress responsivity, with males and females showing distinct patterns of endocannabinoid signaling and receptor expression under stress conditions [14,15]. Females are more sensitive than males to the effects of cannabinoids, and many sexually dimorphic effects can be attributed to a sexually dimorphic ECS [13]. In addition, the ECS contributes to cerebrovascular regulation. Endocannabinoids modulate vascular tone and cerebral blood flow, processes that are particularly relevant under hypoxic stress, when oxygen delivery to the brain is compromised. Craft et al. (2012) reported that ECS activity influences neurovascular coupling, suggesting that alterations in this pathway could contribute to cognitive and affective changes associated with high-altitude exposure [13]. van Doorslaer de Ten Ryen et al. (2023) showed that both endurance and resistance exercise in hypoxia alter the levels of endocannabinoids and the expression of ECS-related enzymes, highlighting a role for ECS signaling in integrating metabolic and vascular responses during physical exertion in oxygen-limited environments [16]. Males have higher CB1 receptor binding sites than females, but females possess more efficient CB1 receptors, and this consistent dimorphism in the ECS may justify the different sensitivity observed between sexes in behavioral paradigms concerning emotion and cognition [17]. Moreover, chronic unpredictable stress exposure results in sexually dimorphic changes in CB1 receptor density, with male rats showing decreased hippocampal CB1 receptor density while female rats exhibit a significant increase following the same stress exposure [18]. Sex differences exist with regional selectivity in the neuroanatomical arrangement of the ECS, and endocannabinoids exhibit sexual dimorphism in serum concentrations across the lifespan [19]. These sex differences may contribute to the differential prevalence and presentation of stress-related psychiatric disorders observed between men and women.

Beyond its classical biochemical regulation, the ECS is also influenced by epigenetic mechanisms, particularly DNA methylation and microRNA (miRNA) regulation, which provide dynamic control over gene expression without altering the underlying DNA sequence [20]. These mechanisms are increasingly recognized as critical mediators of stress adaptation, capable of responding to environmental challenges and potentially predicting individual vulnerability to stress-related disorders [21]. DNA methylation, the addition of methyl groups to cytosine residues of CpG dinucleotides in CpG islands, is a fundamental mechanism in transcriptional repression [22]. MicroRNAs provide a complementary post-transcriptional regulatory mechanism of gene expression: these small non-coding RNAs bind to specific messenger RNAs (mRNAs) to inhibit translation or promote degradation [20]. The epigenetic landscape itself shows sex-specific patterns with males and females often displaying divergent epigenetic responses to the same environmental stressors [23]. Previous research has identified specific miRNAs, including miR-23b-3p (which directly targets *CNR1*) and miR-4505 (associated with psychiatric outcomes), as potential biomarkers in stress-related contexts [24,25,26]. Moreover, coordinated regulation through multiple epigenetic mechanisms—such as simultaneous changes in DNA methylation and miRNA expression [27]—may provide more robust insights into stress adaptation than single-marker approaches.

The present pilot and explorative study investigated epigenetic changes in the *FAAH* and *CNR1* genes in Italian mountaineers participating in a Himalayan expedition. Saliva samples were collected before ascent (T0) and after ascent (T1) from both male and female participants, allowing for sex-stratified analysis of epigenetic responses. Given that several subjects reported psychological stress at T1, and considering the established sexual dimorphism in both stress responses and ECS function, we hypothesized that: (1) high-altitude exposure would induce measurable epigenetic changes in FAAH and *CNR1* genes; (2) these changes would differ between males and females, reflecting sex-specific patterns; (3) epigenetic changes might vary between individuals who maintain psychological resilience and those who experience distress; and (4) directional concordance between DNA methylation and miRNA expression patterns might suggest *CNR1* as a candidate regulatory target in high-altitude-related stress responses.

These preliminary findings may contribute to informing future sex-specific research designs and help generate hypotheses regarding potential epigenetic indicators of psychological vulnerability to extreme altitude activities, pending validation in larger and adequately powered cohorts.

## 2. Materials and Methods

This was a prospective observational longitudinal pilot field study conducted during a trekking expedition in the Khumbu Valley (Nepal). Twenty-one healthy volunteers (14 males, 7 females), all lowlanders without previous acclimatization to high altitude, participated in the study. All subjects provided written informed consent prior to enrolment (Ethics Review Board of the Nepal Health Research Council, ref. no. 1084). Inclusion criteria were age between 18 and 70 years, absence of chronic medical or psychiatric conditions, and fitness for trekking as assessed by a pre-expedition medical examination. Exclusion criteria included history of cardiovascular or pulmonary disease, neurological disorders, or current use of psychotropic medication. Given the logistical and environmental constraints inherent to high-altitude field research, the sample size was determined by feasibility and should be considered exploratory rather than powered for definitive epigenetic inference.

Male participants were older than female participants (mean age 40.8 ± 11.4 vs. 27.9 ± 9.7 years). All female participants were premenopausal at the time of the study (Table 1).

Participants began the trek at Lukla (2860 m) and ascended gradually along the Khumbu Valley to the Everest-K2-CNR Pyramid Laboratory (5050 m). The total ascent was performed over approximately 8–10 days, following standard acclimatization schedules to minimize the risk of acute mountain sickness (AMS). No participants met the criteria for acute mountain sickness (AMS) during the expedition, and no clinically significant AMS symptoms were reported.

All participants completed the trekking expedition as a single group, following the same route and schedule, and progressed together at a comparable pace throughout the ascent. The study comprised two assessment points:

- T0 (baseline): At low altitude (Lukla, prior to ascent).
- T1 (high altitude): During the stay at the Pyramid Laboratory (5050 m). At both T0 and T1, participants completed a battery of validated psychometric instruments:
- SVARAD (for the assessment of irritability and related affective dimensions).
- Beck Depression Inventory (BDI), evaluating depressive symptom severity.
- Zung Self-Rating Anxiety Scale (SAS), assessing state anxiety.
- Snaith–Hamilton Pleasure Scale (SHAPS), measuring hedonic capacity and anhedonia.

All questionnaires were administered in their validated Italian versions. Scoring followed standard procedures, with higher scores reflecting greater symptom severity (for BDI, SAS, SVARAD) or reduced hedonic tone (for SHAPS).

In addition to psychometric testing, saliva samples were collected concurrently at T0 (prior to ascent) and T1 (at the Pyramid Laboratory, before descent) for subsequent molecular analysis. Samples were collected in the morning, after an overnight fast, and stored at controlled temperature. The analyses were subsequently performed at the University of Teramo laboratory. Saliva samples, brought to room temperature, were aliquoted and processed.

Genomic DNA was extracted from 1 mL of saliva using a Salting-Out method, adapted from Garbieri et al. [28]. DNA concentration and purity were assessed using a NanoSNAP™ spectrophotometer (SPEX CertiPrep (Antylia Scientific), Metuchen, NJ, USA). A260/280 ratios near 1.8 were considered acceptable. The methylation status of human *CNR1* and *FAAH* genes was assessed by pyrosequencing of bisulfite-converted DNA, as already described in our previous works [29,30] and detailed in Supplementary Materials. The expression of exosomal salivary miRNAs was assessed by quantitative PCR (qRT-PCR) and detailed in Supplementary Materials.

All data are reported as mean ± SD, and statistical analyses were carried out using GraphPad Prism 9^®^ (Graph-Pad Software, San Diego, CA, USA). For DNA methylation data, differences in CpG site methylation levels between T0 and T1 were assessed using the Wilcoxon signed-rank test, which was selected to account for the paired nature of the study design, as the same individuals were sampled at both time points. To account for multiple comparisons across CpG sites, the Holm-Šídák correction was applied. The application of a paired statistical test for miRNA data was not feasible, as analyses were performed on pooled samples loaded in duplicate, yielding only two technical replicates per group. These replicates do not represent matched individual measurements and therefore do not fulfill the requirements for paired statistical testing. Accordingly, miRNA expression results are presented in a descriptive manner, with bar graphs intended to illustrate the direction and magnitude of expression differences between time points rather than to establish statistical significance. A *p*-value < 0.05 was considered statistically significant for DNA methylation analyses following Holm–Šídák correction. Given the constraints described above, no significance threshold was applied to miRNA expression comparisons.

## 3. Results

### 3.1. Psychometric Scale Results

The combined use of self-administered and clinician-administered scales allowed for a multidimensional assessment of the participants’ psychological conditions. The comparison between T0 and T1 enabled the evaluation of changes in depression, anxiety, anhedonia, and other psychopathological indicators following the climbing experience, with additional exploration of the motivational role of alcohol craving. Mean scores on the main psychometric scales from pre-expedition (T0) to post-expedition (T1) showed an increase, with statistically significant differences observed in exploratory analyses. Specifically, the BDI (*p* = 0.0085, r = 0.574), the SAS (*p* = 0.0067, r = 0.592) and the SHAPS (*p* = 0.0086, r = 0.574) revealed a statistically significant increase, with large effect sizes. Similarly, SVARAD 1 showed a marked increase (*p* = 0.0003, r = 0.781). Similarly, the psychopathological dimensions assessed using the SVARAD showed a marked increase across nearly all items, particularly those related to apprehension/fear (SVARAD 1, *p* = 0.0003), apathy (SVARAD 5, *p* = 0.0049), impulsivity (SVARAD 6, *p* = 0.0006), reality distortion (SVARAD 7, *p* < 0.001), and somatization (SVARAD 9, *p* < 0.001). Desire to drink showed no change (unchanged mean score, *p* = 1.0). The increases were statistically significant using both the *t*-test and the Wilcoxon test, with generally very low *p*-values (<0.01) (Table 2). No formal correction for multiple comparisons was applied to these analyses due to the pilot nature of the study and the limited sample size. Both parametric (paired *t*-test) and nonparametric (Wilcoxon signed-rank test) tests were performed to ensure robustness of the results, given the small sample size and potential deviations from normality.

The changes (Δ) observed between T0 and T1 did not reveal statistically significant differences between females and males on any of the scales considered. A slightly higher mean increase was observed in males for the SAS and SHAPS and in females for the BDI, but none of these differences reached statistical significance (*p* > 0.1–0.8).

### 3.2. miRNA Results

Due to limited RNA yield, miRNA analyses were performed on pooled samples loaded in duplicate. As technical replicates of a single biological observation per group, these data do not permit statistical inference and are presented in a purely descriptive manner to illustrate the direction of expression differences between groups.

**Figure 1:**
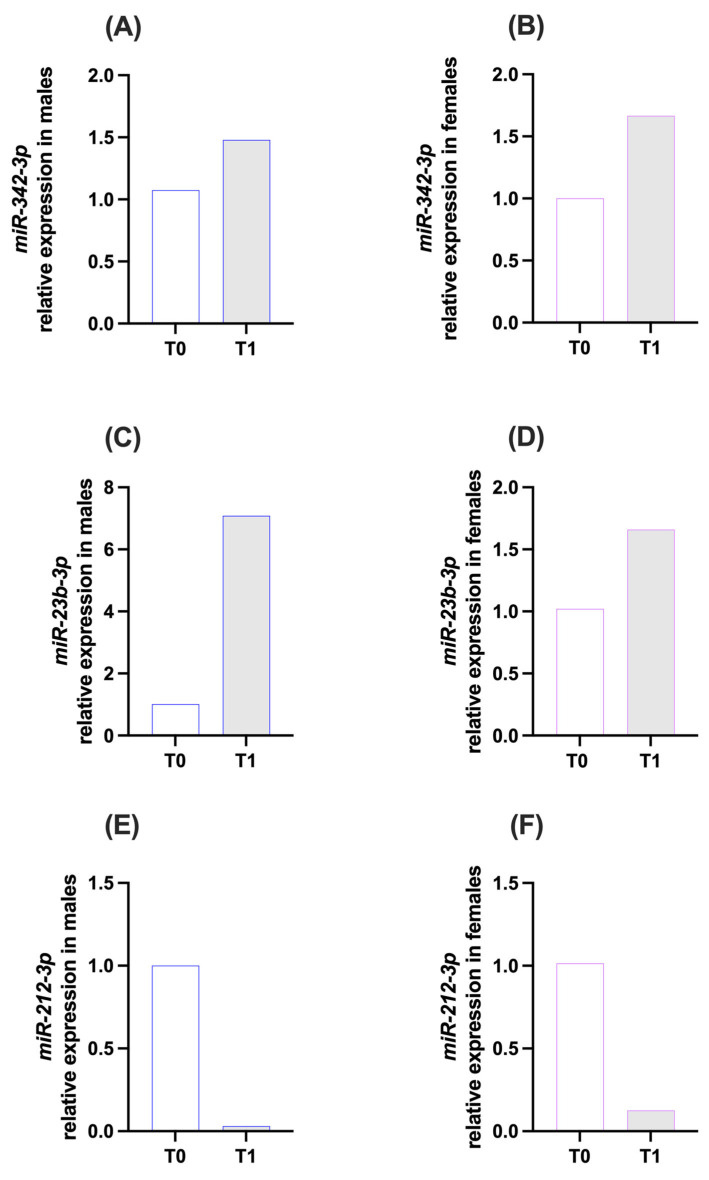
Expression of miR-342-3p (**A**,**B**), miR-23b-3p (**C**,**D**) and miR-212-3p (**E**,**F**), targeting *CNR1*, in saliva samples of the two experimental groups: males and females, at time points T0 (pre-expedition) and T1 (post-expedition). miRNA expression data are reported as 2^−∆∆Ct^ values calculated by the Delta–Delta Ct (∆∆Ct) method versus T0. Expression was normalized to hsa-miR-16-5p. Values are expressed as mean. No statistical test was applied; data are presented in a purely descriptive manner. n = 1 pooled sample per group, loaded in duplicate.

Expression of the *CNR1*-targeting miRNA miR-342-3p showed higher levels at T1 compared to T0 in both males (T0 = 1.08; T1 = 1.48; Figure 1A) and females (T0 = 1.00; T1 = 1.67; Figure 1B). The magnitude of this increase appeared greater in females than in males. miR-23b-3p showed higher expression at T1 compared to T0 in both males (T0 = 1.01; T1 = 7.08; Figure 1C) and females (T0 = 1.02; T1 = 1.66; Figure 1D). In contrast, miR-212-3p showed lower expression at T1 compared to T0 in both males (T0 = 1.00; T1 = 0.03; Figure 1E) and females (T0 = 1.02; T1 = 0.13; Figure 1F). The reduction appeared more pronounced in males.

**Figure 2:**
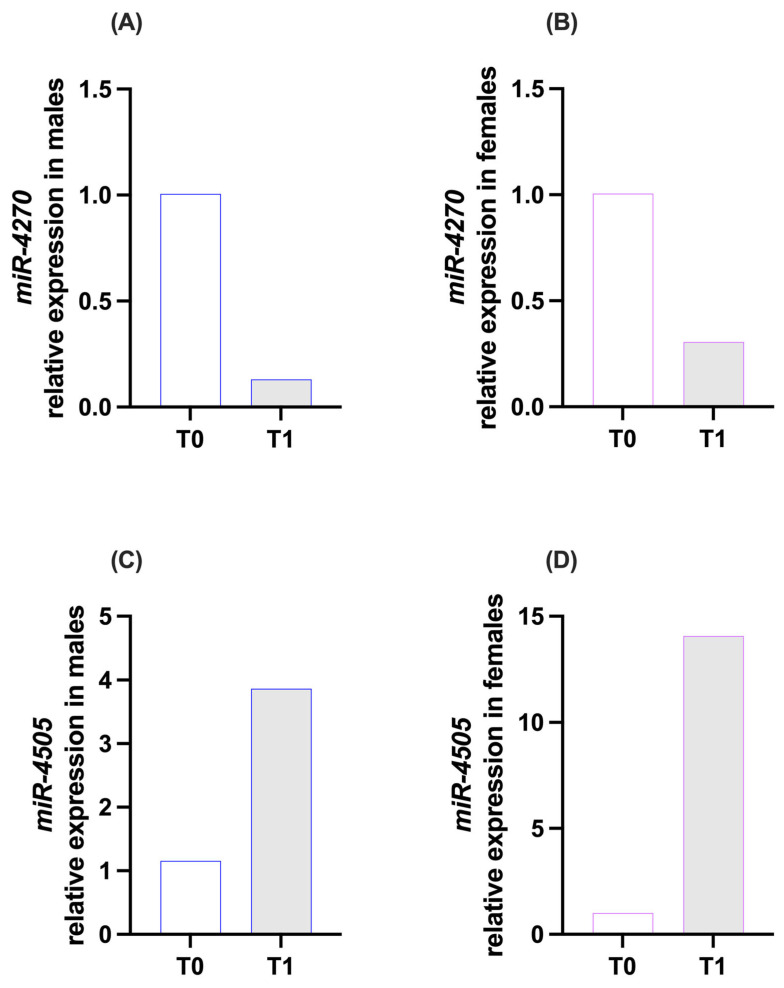
Expression of miR-4270 (**A**,**B**) and miR-4505 (**C**,**D**), targeting FAAH, in saliva samples of the two experimental groups: males and females**,** at time points T0 (pre-expedition) and T1 (post-expedition). miRNA expression data are reported as 2^−∆∆Ct^ values calculated by the Delta–Delta Ct (∆∆Ct) method versus T0. Expression was normalized to hsa-miR-16-5p. Values are expressed as mean. No statistical test was applied; data are presented in a purely descriptive manner. n = 1 pooled sample per group, loaded in duplicate.

Among FAAH-targeting miRNAs, miR-4270 showed lower expression at T1 compared to T0 in both males (T0 = 1.01; T1 = 0.13; Figure 2A) and females (T0 = 1.01; T1 = 0.31; Figure 2B). The reduction appeared more pronounced in males. miR-4505 showed higher expression at T1 compared to T0 in both males (T0 = 1.16; T1 = 3.87; Figure 2C) and females (T0 = 1.01; T1 = 14.08; Figure 2D), with the increase appearing to be greater in females.

All miRNA findings should be considered purely exploratory and require validation in a larger cohort with independent biological replicates.

### 3.3. DNA Methylation Results

**Figure 3:**
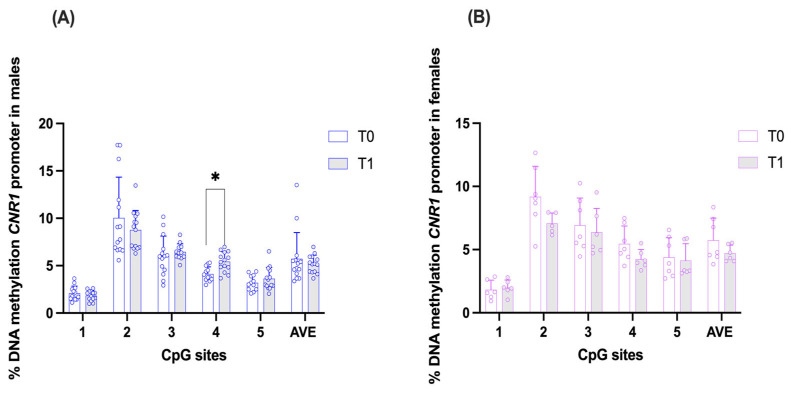
Comparison of DNA methylation status at *CNR1* gene promoter in saliva samples of the two experimental groups: males (**A**) and females (**B**), at time points T0 (pre-expedition) and T1 (post-expedition). Data are expressed as mean ± standard deviation (SD) of the methylation % values of individual CpG sites under study as well as of the average (AVE) of the five CpG sites. Significant differences are indicated: Multiple Paired Wilcoxon signed-rank T-Tests, Holm–Sidak corrected * *p* = 0.037487, n = 12–14 males, n = 6–7 females.

Within the male study population, DNA methylation levels at the *CNR1* gene promoter exhibited minimal temporal variation between the baseline time point (T0) and the follow-up assessment (T1). Specifically, CpG1 showed a slight decrease in methylation, from 2.09 ± 0.75 to 1.84 ± 0.53 (*p* = 0.787115). CpG2 decreased from 10.04 ± 4.29 to 8.77 ± 2.05 (*p* = 0.787115), while CpG3 showed a slight increase from 6.03 ± 2.10 to 6.46 ± 0.85 (*p* = 0.787115). CpG4, however, showed a statistically significant increase in methylation at T1, rising from 4.12 ± 0.76 to 5.42 ± 1.03 (*p* = 0.037, r = 0.556), representing a large effect size. Finally, CpG5 showed a slight increase from 3.20 ± 0.80 to 3.62 ± 1.19 (*p* = 0.787115). The average (AVE) of all five CpG sites under study was slightly lower at T1 (5.17 ± 0.94) compared to T0 (5.72 ± 2.77, *p* = 0.787115), indicating that no major global changes in *CNR1* methylation were observed, although CpG4 showed a specific significant increase after the expedition (Figure 3A).

When data were stratified on the basis of the age of male subjects (>40 years, <40 years, no differences in *CNR1* DNA methylation levels were observed (Supplementary Figure S2).

In female participants, none of the analyzed CpG sites showed statistically significant differences in methylation levels between T0 and T1 (*p* > 0.05 for all CpG sites). More specifically, CpG1 showed a slight increase from 1.82 ± 0.77 to 2.00 ± 0.60 (*p* = 0.963364). CpG2 decreased from 9.19 ± 2.41 to 7.06 ± 0.83 (*p* = 0.46410), while CpG3 showed a small reduction from 6.93 ± 2.15 to 6.38 ± 1.88 (*p* > 0.999999). CpG4 also showed a decrease from 5.46 ± 1.41 to 4.24 ± 0.76 (*p* = 0.551205), and CpG5 from 4.39 ± 1.56 to 4.13 ± 1.34 (*p* > 0.999999). A pattern of general stability in methylation levels was observed, consistent with findings in males. The average (AVE) was slightly lower at T1 (4.71 ± 0.65) compared to T0 (5.74 ± 1.75, *p* = 0.963364), suggesting a modest reduction in overall *CNR1* methylation, although not statistically significant (Figure 3B).

**Figure 4:**
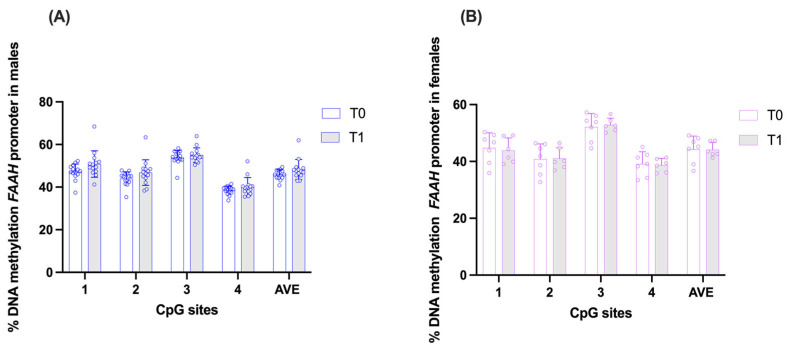
Comparison of DNA methylation status at *FAAH* gene promoter in saliva samples of the two experimental groups: males (**A**) and females (**B**), at time points T0 (pre-expedition) and T1 (post-expedition). Data are expressed as mean ± standard deviation (SD) of the methylation % values of individual CpG sites under study as well as of the average (AVE) of the four CpG sites. Multiple Paired Wilcoxon signed-rank *t*-tests, Holm–Sidak corrected, n = 14 males, n = 6–7 females.

Regarding the *FAAH* gene promoter, in the group of men, a generalized increase in methylation levels was observed at all CpG sites between T0 and T1. The average methylation percentage increased from 45.84 ± 2.31% to 48.22 ± 4.73%, although the difference was not statistically significant (*p* = 0.223930). CpG1 showed an increase from 47.14 ± 3.71 to 50.83 ± 6.21 (*p* = 0.223930), and CpG2 from 43.95 ± 3.24 to 46.83 ± 6.00 (*p* = 0.35); these two sites showed the most marked increases, although still not statistically significant. CpG3 and CpG4 showed non-significant increases, rising from 53.83 ± 3.30 to 55.04 ± 3.43 (*p* = 0.463558) and from 38.42 ± 2.03 to 40.20 ± 4.32 (*p* = 0.463558), respectively. No statistically significant changes were observed across CpG sites (Figure 4A).

When data were stratified on the basis of the age of male subjects (>40 years, <40 years, no differences in *FAAH* DNA methylation levels were observed (Supplementary Figure S3).

In the group of women, no statistically significant changes were observed in *FAAH* methylation levels between T0 and T1 for any CpG site (*p* = 0.943686 for all). Specifically, CpG1 showed a slight decrease from 44.76 ± 5.33 to 43.82 ± 4.47. CpG2 showed a minimal increase from 40.82 ± 5.38 to 41.03 ± 3.79, while CpG3 showed a small increase from 52.11 ± 4.75 to 52.85 ± 2.36. CpG4 showed a marginal reduction from 39.13 ± 4.27 to 38.71 ± 2.35. The average (AVE) of the four CpG sites under study remained essentially unchanged, moving from 44.21 ± 4.69 to 44.10 ± 2.57 (*p* = 0.943686). The general pattern remained stable and consistent across CpG sites, suggesting the absence of relevant methylation changes during the observation period (Figure 4B).

## 4. Discussion

The present pilot and exploratory study investigated epigenetic patterns in the endocannabinoid system in healthy lowlanders exposed to Himalayan high-altitude conditions, examining both DNA methylation and descriptive miRNA expression data for *CNR1* and FAAH genes in relation to psychometric outcomes, with preliminary observations exploring possible sex-related variation. Findings should be interpreted with caution given the preliminary nature of the study, the small sample size, and the exploratory design; they are best understood as hypothesis-generating observations rather than definitive conclusions.

Psychometric assessments indicated that, overall, participants exhibited relatively low levels of clinical symptomatology, although a general decline in mood and hedonic capacity was observed. This pattern is consistent with evidence showing that hypoxia, isolation, and the physiological burden of high-altitude exposure may act as risk factors for transient affective and cognitive disturbances [4]. Some subtle differences emerged in response patterns across scales between males and females, which may point to partially distinct manifestations of stress-induced psychological distress; however, these observations should be interpreted with caution given the limited statistical power of sex-stratified analyses. Analysis of the SVARAD sub-scales, capturing diverse dimensions of stress vulnerability such as emotional reactivity, coping strategies, perceived social support, and emotion regulation [31], revealed some cumulative sex-specific profiles despite the absence of statistically significant individual differences. One possible interpretation is that this reflects sample size limitations rather than a true absence of effect. These patterns are consistent with established evidence of sexually dimorphic stress responsivity [32].

At the molecular level, our data offer exploratory observations consistent with the possibility of epigenetic changes in ECS genes following Himalayan climbing exposure, with exploratory observations suggesting possible sex-related differences that warrant further investigation. The primary robust finding is the statistically significant increase in *CNR1* CpG4 promoter methylation in males at T1, while DNA methylation remained largely stable across all other sites and in females. The ECS’s role in stress resilience is well-documented [33,34,35], and converging preclinical and clinical data suggest that modulation of endocannabinoid signaling influences anxiety-related outcomes [36]. *CNR1* expression and its epigenetic regulation have been implicated in psychiatric disorders, particularly in the context of environmental stress [30,37,38,39], providing a rationale for examining these markers in the context of extreme altitude-related stress.

The specific miRNAs examined in this study were selected based on their predicted and validated regulatory interactions with *CNR1* and FAAH. Among these, miR-23b-3p has been demonstrated to directly bind to the 3′-UTR of *CNR1* [40], miR-212-3p has been implicated in neuropsychiatric contexts and stress responses [24,25,26], and miR-342-3p represents an additional predicted regulator based on bioinformatic analyses. For FAAH, miR-4505 has been associated with psychiatric outcomes in previous research [41,42,43].

Descriptive miRNA expression data showed directional differences between groups for all miRNAs examined. Given that analyses were performed on pooled samples, these observations cannot be statistically evaluated and should be interpreted solely as exploratory.

The DNA methylation analysis of *CNR1* and FAAH promoters revealed relatively stable patterns across most CpG sites, with one notable exception: CpG site 4 in the *CNR1* promoter of males showed a statistically significant increase in methylation at T1. This represents the primary robust finding of the present study. The general stability of DNA methylation across other CpG sites suggests that the acute stress of mountaineering may not induce widespread methylation changes in these genes within the timeframe studied. Speculatively, the observed change at *CNR1* CpG site 4 in males could be consistent with a regulatory response to the climbing challenge that could potentially affect *CNR1* expression and endocannabinoid signaling, though functional validation would be required to confirm this interpretation.

Of potential interest, although the non-significant directional increase in miR-23b-3p expression in males was observed descriptively, the direction of change is consistent with the observed increase in *CNR1* promoter methylation. Both increased miRNA expression and increased promoter methylation would be expected to reduce *CNR1* expression, raising the possibility, to be tested in future adequately powered studies, of a coordinated epigenetic response at the *CNR1* locus in males. This observation is purely speculative given the descriptive nature of the miRNA data but provides a hypothesis for future investigation. The fact that females showed neither significant methylation changes nor consistent directional changes in miR-23b-3p represents an exploratory observation that may hint at possible sex-related differences in epigenetic stress responses, though the limited female sample size (n = 7) and the descriptive nature of the miRNA data preclude any interpretive conclusion. The ECS exhibits known sexual dimorphism at multiple levels, including receptor density and circulating endocannabinoid levels [13,17] and our results, though do not contradict prior evidence of sexual dimorphism in the ECS, are insufficient to draw any conclusion regarding sex-specificity of epigenetic stress responses.

Substantial inter-individual variability was observed within each sex group in psychological outcomes. This variability likely reflects the interaction of multiple factors, including genetic background, baseline epigenetic profiles, neuroendocrine differences, and psychological coping strategies, all of which represent important directions for future investigation.

We acknowledge that the 13-year mean age difference between male (40.8 years) and female (27.9 years) participants represents a potential confounding factor in the interpretation of sex-related differences in *CNR1* methylation at CpG4. To assess the potential influence of age on DNA methylation patterns, we performed an age-based stratification analysis in the male group, defining two subgroups: participants under 40 years of age and participants over 40 years of age. No significant differences in methylation profiles were observed between the two male age subgroups, suggesting that, within the present sample, age did not substantially confound the epigenetic patterns described. Age-based stratification was not feasible for the female group due to the highly skewed age distribution: with the exception of one participant aged 49 years, all other female participants were between 20 and 29 years of age. Consequently, the possibility that the observed sex difference in *CNR1* CpG4 methylation is at least partially attributable to the age disparity between groups cannot be entirely excluded. This finding should therefore be interpreted with caution and considered hypothesis-generating, pending replication in larger, age-matched cohorts. Notably, all female participants were premenopausal at the time of the study, ensuring a degree of hormonal homogeneity within the group and mitigating concerns related to menopausal transition as a potential confounding factor.

## 5. Limitations of the Study

Several limitations should be acknowledged when interpreting the present findings. The relatively small sample size, particularly after sex stratification (n = 14 males, n = 7 females), limits the statistical power and restricts the ability to detect small-to-moderate effect sizes. Sex-specific observations should not be interpreted in mechanistic or generalizable terms, and replication in larger, sex- and age-balanced cohorts is warranted. The inability to perform age-based stratification in the female group means that age-related methylation drift cannot be fully excluded as a contributing factor. The use of saliva as a biological matrix represents an indirect proxy of brain-related epigenetic processes, and changes in DNA methylation may partly reflect shifts in cellular composition rather than true epigenetic remodeling. miRNA analyses were performed on pooled samples, precluding individual-level biological inference; findings are therefore purely descriptive. A further limitation concerns the selection of miR-16-5p as the reference gene for data normalization. Although miR-16-5p expression was empirically verified to be uniform across all samples and time points in the present cohort, we acknowledge that its expression can be influenced by hypoxic conditions in certain biological contexts. Future studies should consider a more systematic validation of reference gene stability using dedicated algorithms (e.g., geNorm, NormFinder) applied to a panel of candidate reference genes. The absence of post-expedition follow-up sampling prevented the assessment of the temporal stability of the observed epigenetic changes. No direct measurements of ECS functional outputs were performed, and whether the observed epigenetic modifications translate into functionally relevant changes in ECS activity remains to be established. Finally, the naturalistic field design does not allow for the isolation of hypoxia as the sole determinant of the observed changes, as participants were simultaneously exposed to multiple interacting stressors including physical exertion, sleep fragmentation, cold exposure, and psychological pressure. Taken together, these limitations highlight the need for future studies with larger, more controlled cohorts to validate and extend the present findings.

## 6. Conclusions

Although these findings are preliminary and exploratory, this study contributes early field-based evidence on epigenetic changes in the endocannabinoid system under real-world extreme environmental conditions, with preliminary observations suggesting possible sex-related differences that warrant further investigation.

The primary contribution of this study lies in providing initial field-based evidence that high-altitude related stress may be associated with epigenetic changes at the *CNR1* locus, with exploratory observations of possible sex-related patterns, and in establishing a conceptual and methodological framework for future adequately powered investigations.

## Figures and Tables

**Table 1:** Demographic characteristics of participants (n = 21), including age distribution expressed as mean ± SD, median (IQR), and range, overall and stratified by sex (males and females).

Variable	Total (n = 21)	Males (n = 14)	Females (n = 7)
Age, mean ± SD (years)	36.5 ± 12.7	40.8 ± 11.4	27.9 ± 9.7
Age, median (IQR)	36 (28–50)	46 (28–52)	25 (22–29)
Age range (years)	20–56	22–56	20–4

**Table 2:** Comparison of mean scores (±SD) on the different psychometric scales before (T0) and after (T1) the Himalayan expedition. *p*-values were obtained using *t*-tests and Wilcoxon tests. Statistically significant differences are indicated as *p* < 0.01. ** *p* < 0.01; *** *p* < 0.001.

Variable	T0 Mean ± SD	T1 Mean ± SD	*p*-Value *t*-Test	*p*-Value Wilcoxon
BDI (0–63)	5.86 ± 3.53	6.81 ± 3.72	0.003565 **	0.008531 **
SAS (1–80)	27.90 ± 7.08	30.62 ± 6.28	0.007533 **	0.006680 **
SHAPS (0–14)	2.76 ± 1.18	3.38 ± 1.43	0.003737 **	0.008565 **
SVARAD 1 (0–4)	0.81 ± 0.68	1.71 ± 0.78	0.000009 ***	0.000347 ***
SVARAD 2 (0–4)	0.62 ± 0.59	1.19 ± 0.60	0.000947 ***	0.002700 **
SVARAD 3 (0–4)	0.52 ± 0.60	1.48 ± 0.81	0.000009 ***	0.000317 ***
SVARAD 4 (0–4)	0.71 ± 0.64	1.48 ± 0.75	0.000198 ***	0.001223 **
SVARAD 5 (0–4)	0.57 ± 0.75	1.14 ± 0.48	0.002213 **	0.004897 **
SVARAD 6 (0–4)	0.62 ± 0.59	1.48 ± 0.81	0.000027 ***	0.000565 ***
SVARAD 7 (0–4)	0.00 ± 0.00	1.38 ± 0.59	0.000000 ***	0.000053 ***
SVARAD 8 (0–4)	0.00 ± 0.00	0.00 ± 0.00	1.000000	1.000000
SVARAD 9 (0–4)	0.38 ± 0.67	1.62 ± 0.74	0.000000 ***	0.000069 ***
SVARAD 10 (0–4)	0.48 ± 0.51	2.00 ± 1.18	0.000008 ***	0.000365 ***
Desire to drink (1–5)	1.52 ± 0.68	1.52 ± 0.93	1.000000	1.000000

## Data Availability

The original contributions presented in this study are included in the article. Further inquiries can be directed to the corresponding author.

## Supplementary Materials

The following supporting information can be downloaded at: https://www.mdpi.com/article/10.3390/brainsci16050500/s1, Table S1. Details of sequences and primers employed for the DNA methylation analysis by pyrose-24 quencing; Figure S1. Schematic representation of human (A) *CNR1*(Transcript Cnr1-203, ENST00000369501.3) and (b) FAAH (transcript FAAH-201 ENST00000243167.9) promoter and the 5 upstream region; Figure S2. Comparison of DNA methylation status at *CNR1* gene promoter in saliva samples of (A) male subjects under 40’ and (B) male subjects over 40’ years at time points T0 (pre-expedition) and T1 (post-expedition); Figure S3. Comparison of DNA methylation status at *FAAH* gene promoter in saliva samples of (A) male subjects under 40’ and (B) male subjects over 40’ years at time points T0 (pre-expedition) and T1 (post-expedition).
