# Supplementary material for: Sex-Specific Epigenetic Patterns in Endocannabinoid System Genes Following High-Altitude Exposure: An Exploratory Study
Source: Brain Sci. 2026 May 2;16(5):500. doi: 10.3390/brainsci16050500 (PMC13205093; doi:10.3390/brainsci16050500)
Supplement: Supplementary file 1 [file brainsci-16-00500-s001.zip › brainsci-4201044-supplementary.pdf]

SUPPLEMENTARY MATERIALS AND METHODS

Bisulfite conversion was performed with the EZ DNA Methylation-Gold™ Kit (Zymo Research Orange, CA, USA), according to the manufacturer's protocol. All samples, including both T0 and T1 time points, were processed under identical experimental conditions and in an interleaved manner to prevent batch-related systematic bias. Conversion efficiency was not formally quantified as a standalone quality control measure; however, given the paired within-subject design of the study, any residual non-systematic variability in conversion efficiency would be expected to affect T0 and T1 measurements proportionally, thereby unlikely to introduce a directional bias in the observed methylation differences. A total of 500 ng bisulfite converted genomic DNA for sample was amplified using the PyroMark PCR Kit (QIAGEN, Hilden, Germany) with a biotinylated primer, following the manufacturer's recommendations. PCR conditions were as follows: 95∘C for 15 min, followed by 45 cycles of 94∘ C for 30 s, 56∘C for 30 s, 72∘C for 30 s, and, finally, 72∘C for 10 min. PCR products were verified on a 1.8% agarose gel. DNA methylation levels were quantified by pyrosequencing, using PyroMark CpG Assays (QIAGEN, Hilden, Germany) on the PyroMark Q48 Autoprep system, using PyroMark® Q48 Advanced CpG Reagents (QIAGEN, Hilden, Germany). Primers for the PCR amplification and sequencing of human *FAAH* gene were designed using PyroMark Assay Design Software version 2.0 (Qiagen, Hilden, Germany) to target four CpG sites within the gene's regulatory region. A specific PyroMark CpG assay (Qiagen, Hilden, Germany) was instead employed to analyze five CpG sites in the human *CNR1* gene regulatory region. Detailed informations on the sequences and assays are reported in Table S1 and Figure S1.

Table S1. Details of sequences and primers employed for the DNA methylation analysis by pyrosequencing. Hs= human; CpG = C-phosphate-G; F = forward primer; Biot_R = biotinylated reverse primer; S = sequencing primer. Bold text = CpG sites analyzed.

Gene	Sequence Analyzed	n CpG sites	5'-primers-3'
Hs_CNR1	**cg**tttggaaatactttttc**cg**cctccc**cg**ccccct**cg**ggta**cg**a	5	Included in the Qiagen PyroMark CpG Assay Hs_CNR1_02_PM PM00122038
Hs_FAAH	gt**cg**g**cg**g**cg**ggtctg**cg**at	4	F: tggattaggagtatagtagatattttg<br>Biot_R: acatccactactatttactacca<br>S: ggatttagagggatggaa

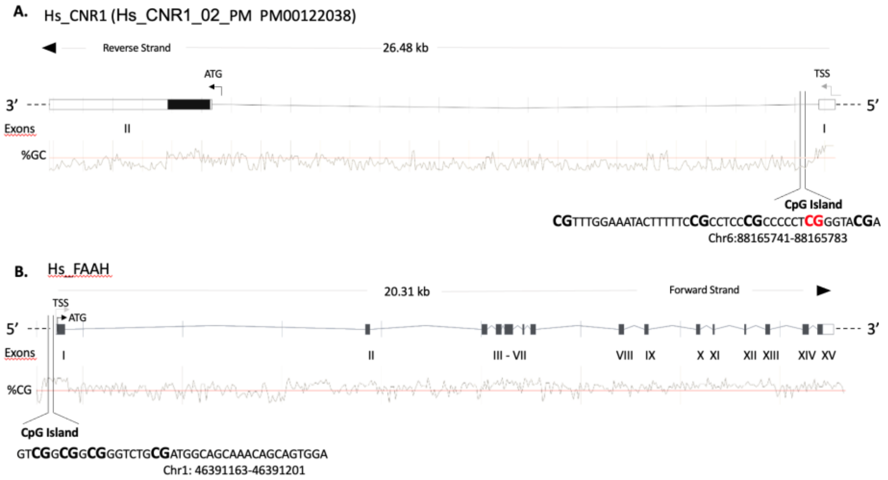

Figure S1. Schematic representation of human (A) CNR1(Transcript Cnr1-203, ENST00000369501.3) and (b) FAAH (transcript FAAH-201 ENST00000243167.9) promoter and the 5′ upstream region. ATG is the translation start site. Shown are the location of CpG island, exon and TSS (transcription

start site). Coding regions of exons are shown darker. Sequence of CpG islands (CNR1 GRCh38 Chr6:88165741-88165783, FAAH GRCh38 Chr1: 46391163-46391201) used for DNA methylation analyses is also reported. Bold text indicates the CpG sites analyzed.

DNA methylation level was analyzed through the PyroMarkQ48 Autoprep 2.4.2 software which calculates the methylation percentage mC/(mC + C) (mC=methylated cytosine, C=unmethylated cytosine) for each CpG site, allowing quantitative comparisons. Quantitative methylation results were expressed both as percentage of every single CpG site and as the average of the methylation percentage of all the CpG sites under study.

Exosomes were isolated from 1 ml of saliva using the Total Exosome Isolation Reagent (Invitrogen™ by Thermo Fisher Scientific, Vilnius, Lithuania). Exosomal RNA was extracted using the Total Exosome RNA and Protein Isolation Kit (Invitrogen™, by Thermo Fisher Scientific, Vilnius, Lithuania) according to the manufacturer's protocol. RNA was eluted in RNase-free water, quantified spectrophotometrically, and stored at −80 °C. Reverse transcription was performed using the miRCURY LNA RT Kit (QIAGEN, Hilden, Germany) with 2–10 ng of RNA in 10 µL reactions (42 °C for 60 min, followed by 95 °C for 5 min). qRT-PCR reactions were run on a QIAquant 96 system (QIAGEN, Hilden, Germany) using SensiFAST™ SYBR® Lo-ROX Kit (Bioline reagents, London, GB) and specific miRCURY LNA miRNA PCR Assays (QIAGEN, Hilden, Germany). Due to limited RNA yield, cDNA samples were pooled by experimental group. Five miRNAs were selected based on predicted interactions with *CNR1* and *FAAH*. miRNAs targeting *CNR1* and *FAAH* were identified via three databases: miRbase [1], miRDB [2] and miRanda[3]. Based on this findings, three miRNAs (has-miR-342-3p miRCURY LNA miRNA PCR Assay YP00205625, hsa-miR-23b-3p miRCURY LNA miRNA PCR Assay YP02119314, hsa-miR-212-3p miRCURY LNA miRNA PCR Assay YP00204170) targeting *CNR1* and two (miR-4270 miRCURY LNA miRNA PCR Assay YP02114586) targeting *FAAH* were selected. The level of microRNAs was normalized using hsa-miR-16-5p (miRCURY LNA miRNA PCR Assay YP00205702, QIAGEN), as endogenous control. Relative expression levels were calculated using the ΔΔCt method [4].

Supplementary References

1. A. Kozomara, M. Birgaoanu, and S. Griffiths-Jones, "MiRBase: From microRNA sequences to function," Nucleic Acids Res., vol. 47, no. D1, 2019, doi: 10.1093/nar/gky1141.
2. Y. Chen and X. Wang, "MiRDB: An online database for prediction of functional microRNA targets," Nucleic Acids Res., vol. 48, no. D1, 2020, doi: 10.1093/nar/gkz757.
3. D. Betel, A. Koppal, P. Agius, C. Sander, and C. Leslie, "Comprehensive modeling of microRNA targets predicts functional non-conserved and non-canonical sites," Genome Biol., vol. 11, no. 8, 2010, doi: 10.1186/gb-2010-11-8-r90.
4. K. J. Livak and T. D. Schmittgen, "Analysis of relative gene expression data using real-time quantitative PCR and the 2-ΔΔCT method," Methods, vol. 25, no. 4, pp. 402–408, 2001, doi: 10.1006/meth.2001.1262.

SUPPLEMENTARY FIGURES

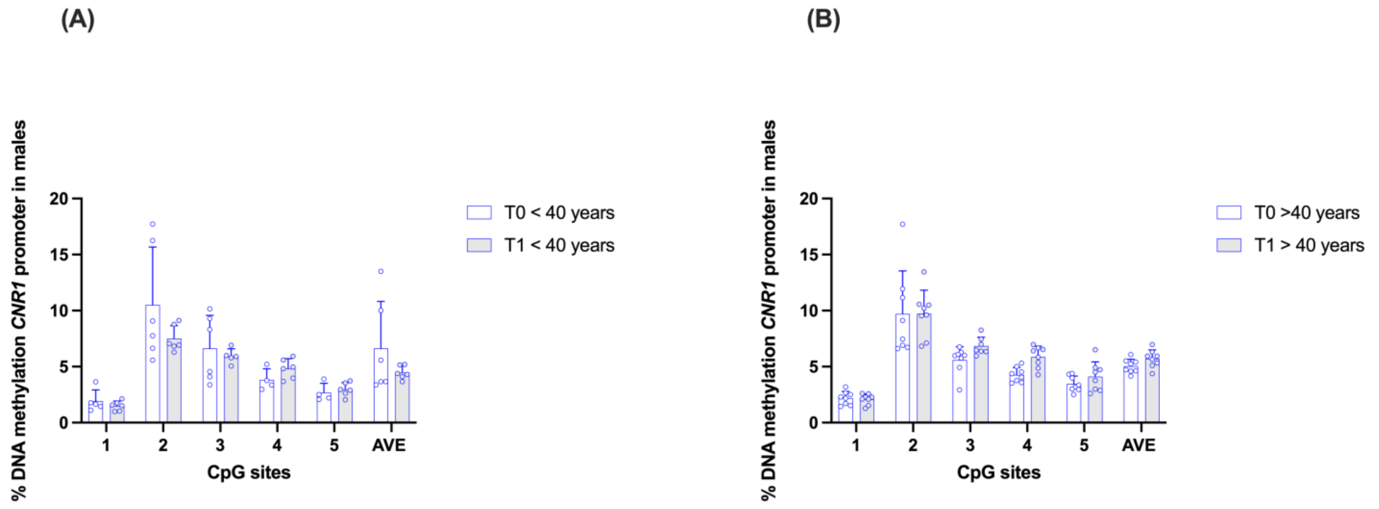

Figure S2 Comparison of DNA methylation status at *CNR1* gene promoter in saliva samples of (A) male subjects under 40′ and (B) male subjects over 40′ years at time points T0 (pre-expedition) and T1 (post-expedition). Data are expressed as mean ± standard deviation (SD) of the methylation % values of individual CpG sites under study as well as of the average (AVE) of the four CpG sites. Multiple paired Wilcoxon t-tests, Holm-Sidak corrected, n=4-6 <40years, n=7-8 >40years.

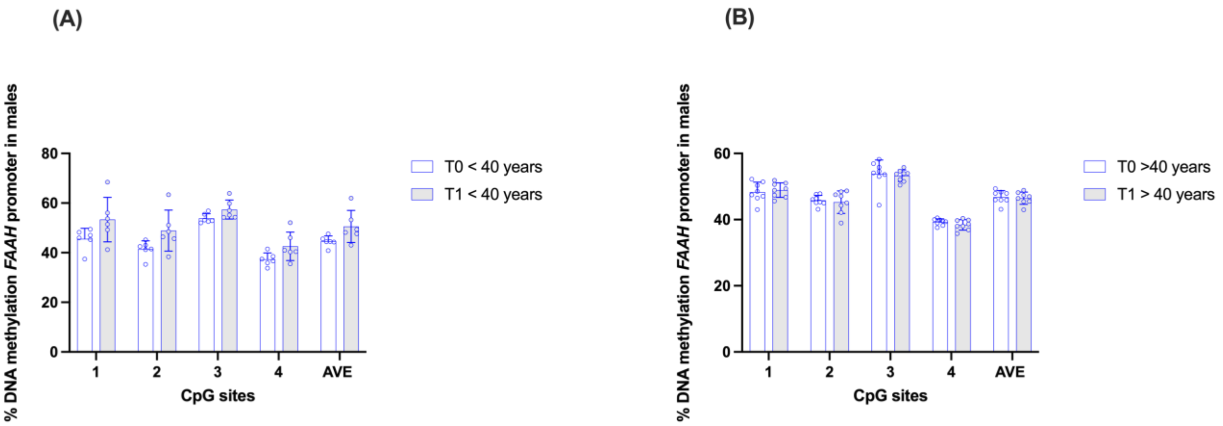

Figure S3 Comparison of DNA methylation status at *FAAH* gene promoter in saliva samples of (A) male subjects under 40′ and (B) male subjects over 40′ years at time points T0 (pre-expedition) and T1 (post-expedition). Data are expressed as mean ± standard deviation (SD) of the methylation % values of individual CpG sites under study as well as of the average (AVE) of the four CpG sites. Multiple paired Wilcoxon t-tests, Holm-Sidak corrected, n=6 <40years, n=8 >40years.
